# Fostering moral reflectivity in community pharmacists through moral case deliberation using the dilemma method

**DOI:** 10.1007/s11096-024-01854-3

**Published:** 2025-01-04

**Authors:** M. Kruijtbosch, A. Floor-Schreudering, E. van Leeuwen, M. L. Bouvy

**Affiliations:** 1 SIR Institute for Pharmacy Practice and Policy, Theda Mansholtstraat 5B, 2331 JE Leiden, The Netherlands; 2 Division of Pharmacoepidemiology and Clinical Pharmacology, Department of Pharmaceutical Sciences, Utrecht University, PO Box 80082, 3508 TB Utrecht, The Netherlands; 3 Department of Primary and Community Care, Radboud University Medical Center, Radboud Institute for Health Sciences (RIHS), 148 RIHS, P.O. Box 9101, 6500 HB Nijmegen, The Netherlands

**Keywords:** Education, Ethics, Morals, Pharmacists, Reflective thinking

## Abstract

**Background:**

Moral case deliberation has been successfully implemented in multidisciplinary groups of secondary care professionals to support ethical decision making. It has not yet been reported for community pharmacists.

**Aim:**

This study investigated whether moral case deliberation fosters moral reflectivity in community pharmacists.

**Method:**

Two moral case deliberations with 14 community pharmacists were guided by two facilitators. One session was described and illustrated with participants’ quotes, detailing each reflection step of the method. An adapted version of the Maastricht evaluation questionnaire was used to understand the effects of the moral case deliberation on participants’ moral reflectivity skills both quantitatively and qualitatively.

**Results:**

In a 2-h session, pharmacists reflected on a moral dilemma concerning double anticoagulant therapy of one presenter pharmacist. Participants discussed the pros and cons of two potential actions: dispensing the medication as prescribed without contacting the patient or contacting the patient first. Deliberation highlighted the importance of understanding the patient’s perspective, leading the presenter and two others to shift towards the latter action.

The evaluation questionnaire revealed that all 14 participants felt supported by the deliberation and the facilitator in recognising the dilemma’s moral dimension and understanding their own and others’ values behind arguments and how these influenced different perspectives. They all felt encouraged to critically reflect, to ask open questions and to delay judgements. The method helped all to morally justify their final decision, with six participants arriving at a decision different from their initial perspective towards the dilemma’s resolution.

**Conclusion:**

This study demonstrates that moral case deliberation enables pharmacists to critically examine their reasoning and reach morally sound resolutions, supporting pharmacists’ professionalism and ethical decision-making.

**Supplementary Information:**

The online version contains supplementary material available at 10.1007/s11096-024-01854-3.

## Impact statements

- The dilemma method, guided by a trained facilitator, effectively teaches community pharmacists’ moral reflectivity skills for wisely resolving moral dilemmas in pharmaceutical care.
- Moral case deliberation enables pharmacists to compare moral perspectives on patient care, fostering reorientation of their routines to improve future professionalism.

## Introduction

Most health professionals experience moral dilemmas on a daily basis. Moral dilemmas are characterized by a choice between options neither of which is a clear ‘best’ course of action: there always will be a conflict between the values and perspectives of the different parties involved (the patient, other health professionals, institutions, society) [1]. Moral dilemmas may cause moral distress in health professionals, which subsequently may negatively affect the quality of care they provide [2, 3]. Community pharmacists experience a diversity of moral dilemmas [4]. Even the best scientific evidence, clinical experience and critical thinking do not provide a sufficient basis for deciding what to do in such dilemmas. It needs the ability to reflect on the values and perspectives of all parties involved, especially of the patient. Dealing with moral dilemmas thus requires clinical and ethical competences for good clinical decision making [5]. In medical professions, moral case deliberation (MCD) is increasingly used to teach such ethical competences [5–8].

MCD is a structured conversation in which answers to a moral question in the context of a participant’s in real practice experienced dilemma case is jointly examined by a group of health professionals under the guidance of a trained facilitator [6]. This method is characterized by participants’ systematic moral reflections (i.e. value judgments in relation to the dilemma) and continuous dialogical understanding (i.e. continuous listening and being open to what others say and trying to see the point they make or understand the arguments they give) in several methodical steps [9, 10].

Research has shown that moral case deliberation may improve moral competences, reduce moral distress, stimulate collaboration among health professionals, and enhance quality of care [3, 7, 11]. As far as we know, it has only been evaluated among professionals working in multidisciplinary teams in hospitals and long-term care institutions [6, 8, 10, 12], and within care for specific patient groups like elderly care [11, 13], mental healthcare [12] and care for the homeless [14]. MCD outcomes as perceived by participants working in such teams point in the direction of i.e. enhanced emotional support and collaboration, improved moral reflexivity, improved moral attitude, as well as concrete case results [15–17]. Moral dilemmas in such multidisciplinary institutions mostly do not need immediate action, which allows for reflection time with a team of professionals and other relevant parties.

Pharmacists’ dilemmas in community pharmacy often happen with the patient waiting at the counter and thus need quick settlement. This leaves limited possibilities for extensive moral deliberation with colleagues at the time of the dilemma. MCD may stimulate the moral competences necessary for community pharmacists. Although in some countries systematic step-wise decision-making approaches are available, targeted at the self-learning of individual pharmacists and pharmacy students [18, 19], moral case deliberation is not well established for community pharmacists. It is therefore interesting to explore what outcomes can be distinguished in community pharmacists when such deliberation is performed.

### Aim

The aim of this study was to explore whether moral case deliberation fosters moral reflectivity required for community pharmacists to make ethical decisions in moral dilemma cases.

### Ethics approval

This study is compliant with the requirements of the institutional review board of the division of Pharmacoepidemiology and Clinical Pharmacology at Utrecht University. The board had no objection to the conduct of the study, declared on 4th March 2020 under code UPF2001. All participants consented to the use of the recording of the deliberation for the study. Data that could give clues about the origin of dilemma cases (e.g. names of patients, cities, pharmacies, pharmacists or physicians) were removed.

## Method

### Study design

A descriptive study of the effects of moral case deliberation on community pharmacists’ moral reflectivity using the dilemma method [20, 21].

### Case presenters, participants, facilitators

One pilot MCD session and two subsequent sessions for the research required three distinct case presenters. In the Netherlands, the postgraduate pharmacist specialization program incorporates a professionalism curriculum, mandating early-career community pharmacists to document a moral dilemma encountered in practice. Community pharmacists who recently had graduated from this program were contacted by e-mail with the invitation to become presenters of their dilemma cases in the three deliberation sessions of this study. The first three pharmacists who accepted the invitation were selected as presenters and contacted in advance for preparation.

A minimum of five participants, in addition to a presenter, is recommended for an MCD session [6]. Participants for three deliberation sessions were recruited through the presenters' networks and the Utrecht Pharmacy Practice network for Education and Research's electronic newsletter [22]. All the pharmacists who finally agreed to participate were community pharmacists and were therefore beyond the level of the specialization program. The pilot participants were informed that their session was for process evaluation only, not data collection, and those outcomes were not utilized in this study.

Two facilitators (MK and EvL), both members of the research team, facilitated the MCD sessions. MK is a sociologist with a pre-master in applied ethics and a trained MCD-facilitator in the dilemma method [23]. She has 18 years’ work experience in pharmacy practice research. EvL is an experienced senior MCD-facilitator for more than 20 years and emeritus professor in medical ethics. AF and MB are pharmacists with a long experience in pharmacy practice research. To ensure consistency, MK summarized the aims and procedures of each step in a facilitator manual for both MCD sessions.

### Moral case deliberation

In a moral case deliberation, participants engage in systematic exploration of a dilemma case by critically reflecting on each other's moral viewpoints [10]. They may reach consensus on the appropriate resolution, though it is not mandatory. If no consensus is achieved, the process may generate new questions, shedding light on the implications of various perspectives for addressing similar situations [10, 24]. Ultimately, participants' ability to navigate moral dilemmas in practice stands to improve through this collaborative and reflective approach [12, 24].

This study employed the dilemma method [10, 14] as the structure for conversation. This established method focuses on the handling of the dilemma, at least two mutually exclusive options, and finding moral justifications for each such option [10, 14]. The method comprises 10 methodical steps, each serving a specific purpose (see Table 1). These steps beyond the introduction (step 1) are organised into three phases [10]: (1) the image-formation phase (steps 2–6), (2) the judgment-formation phase (step 7) and (3) the decision-making phase (steps 8 and 9). The facilitator's role is to activate participants' moral skills while avoiding interference with the content of the dilemma case [3]. This involves stimulating deliberation by encouraging reflection, posing open-ended questions, and fostering attentive listening among participants. The facilitator challenges presuppositions and biases through critical questioning, prompting participants to substantiate assumptions with moral arguments. Transparency of participants' underlying values is ensured, making them meaningful in the context of the dilemma case.

**Table 1:** Dilemma method: three phases and 10 methodical steps [10]

Phase	Methodical step
	Step 1—Introduction
	*Aim*: To introduce the aim and procedure of MCD; to clarify the roles of facilitator and case presenter (presenter); to explain the manner of conversation (e.g. open communication and questioning, delaying of and providing moral arguments for judgments, listening to others); to emphasize the confidentiality of the meeting
1 Image-formation	Step 2—Presenting the case
	*Aim*: To focus on the experiences of the presenter who presents the dilemma case. The presenter is asked to share the moment of the case in which he/she experienced the moral dilemma most strongly
	Step 3—Formulating the moral question and action options
	*Aim*: To make explicit the moral question underlying the moral dilemma (i.e. the question the presenter wants to answer in the MCD session) as well as the action options the presenter saw at the time of the dilemma, the negative consequences of these action options and the (professional) values involved
	Step 4—Asking clarification questions
	*Aim*: To clarify everything that is still unclear in the situation of the moral dilemma by the presenter. To (re)construct the situation in order to investigate the moral dilemma. Also, to check if the moral question is still accurate
	Step 5—Analysing the case in terms of perspectives, values and norms
	*Aim*: To investigate the moral perspectives of all relevant parties involved in the dilemma. To formulate these parties’ values and a normative rule of action (a norm) that stems from each value
	Step 6—Brainstorming alternative action options
	*Aim*: To jointly brainstorm alternative action options on top of the options already mentioned by the presenter in step 4
2 Judgment-formation	Step 7—Making an individual choice of action and making explicit one’s moral considerations
	*Aim*: To formulate participants’ personal and professional views on the dilemma case. The participants answer the following five questions: a. It was morally justified that I choose action option…(A, B or alternative) b. Because of…(which value or norm)? c. Despite…(which value or norm)? d. How can you limit the negative consequences of your choice mentioned under (c)? e. What do you need to do, according to your answer under (a)?
3 Decision-making	Step 8—Conducting dialogical inquiry on similarities and differences
	*Aim*: To examine the similarities and differences in moral judgments, considerations and values between the participants as formulated in step 7. The focus is on understanding one’s own and others’ viewpoints
	Step 9—Concluding (harvesting)
	*Aim*: To conclude the findings of the MCD session. In prospective cases, the aim may also be to make a plan of action or communicating how the dilemma will be handled and by whom
	Step 10—Evaluating
	*Aim*: To jointly evaluate the MCD session, including the dialogue, atmosphere, results and facilitator

Due to COVID-19 restrictions on physical meetings, Microsoft Teams videoconferencing was utilized for both MCD sessions.

### Data collection

Initially, a pilot MCD session with four community pharmacists was conducted with MK as the facilitator and EvL and AF as observers. All participants received descriptions of pharmacists' professional core values [25] beforehand (Supplementary material). The pilot revealed a need to enhance dialogue in steps 1–3. There was a delay or long gaps in response time among participants, likely influenced by the videoconferencing environment. To address this, the facilitator intensified Socratic questioning in these steps, i.e. prompting participants with open questions or asking further questions so that participants are activated to explain and clarify meaning to what they have said. Such questioning enables participants to shed more light on their own thoughts and implicit knowledge that then becomes transparent and available to others. Subsequent adjustment was made in the two following MCD sessions. These sessions were moderated by one facilitator and observed by another (MK or EvL), recorded and verbatim transcribed (by MK).

In order to be able to understand the effects of the deliberation sessions on participants’ moral reflectivity quantitatively as well as qualitatively, the participants anonymously filled in an online evaluation questionnaire. This questionnaire was based on one existing questionnaire available at the time of the research, the Maastricht evaluation questionnaire [12]. The latter was adapted to the community pharmacy practice context. The questionnaire contained closed questions for example about the effect of the MCD on the participants’ awareness of their own values and arguments and those of other participants. Such questions could be answered with ‘Yes’ or ‘No’. Further, the questionnaire contained statements regarding the role of the facilitator and participants as well as statements regarding the potential of MCD to improve ethical competences. These statements were to be scored on a 4-point scale (i.e. strongly disagree; disagree; agree; strongly agree). Besides, three open questions were asked in the questionnaire to make the participants clarify why to them the deliberation was useful or not useful, to describe what they had learned during the session and, in the case they had answered the question ‘Have you changed your vision on how to approach the dilemma case due to the MCD session?’ with a ‘Yes’, to clarify what had changed in their vision and why.

### Analysis

The deliberation within each reflection step of the dilemma method was described and summarised for one of the two MCD sessions by MK, based on the transcribed text. For the summary of this dilemma case see Table 2. We selected quotes from participants from the transcribed session to illustrate the effects on participants’ views and reasoning in the context of the dilemma case. The results from the online questionnaire were analysed in Excel for the closed questions. The answers given by the participants to the three open questions were viewed by MK, MB and AF. Through consensus in a joint session, quotes were selected and attached to quantitative questions in cases where quotes were considered relevant illustrations in the context thereof.

**Table 2:** Summary of Moral dilemma case 1, case presenter MCD1

A 74-year-old man, recently discharged from a rehabilitation center, was prescribed two antithrombotics (dabigatran twice a day 110 mg and acetylsalicylic acid 80 mg daily). The man's wife, concerned about her husband's medication, approached the pharmacist for advice. The surgeon and geriatrician who prescribed the medications had not explained the reasons behind the prescriptions. The wife worried because her husband had blue bruises and was restless and anxious
The pharmacist couldn't clarify the indication for the double antithrombotic therapy. The geriatrician had continued the therapy without knowledge of the indication, and the surgeon was unavailable. Additionally, the patient's renal function was unknown, preventing the pharmacist from providing advice on the double anticoagulant therapy

This study followed scientific standards for describing qualitative data [26, 27].

### Extra post-study analysis

At the time of the present study one instrument—the European Moral Case Deliberation Outcomes Instrument (Euro-MCD)—was available. The study presenting that instrument showed some insights in what effects MCD could possibly have on participants’ reflectivity skills [15–17]. Those effects were collected specifically among health professionals working in multidisciplinary care institutions, and described in several outcome domains. However, at the time the present research was in development, those outcome domains themselves had not been tested yet in the field among MCD users to gather their perspectives. Additionally, the empirical findings had not undergone normative evaluation, leaving uncertainty about what MCD outcomes should be based on its goals [16]. Hence those outcome domains were not validated and we considered it premature to use the Euro-MCD instrument for the evaluation of the effects of the two MCD sessions of the present study. Since the validation of the instrument (i.e. Euro-MCD 2.0) [28] was published just after the present study was conducted, its outcome domains were utilized for interpretation of the results in the discussion section.

The Euro-MCD 2.0 includes 15 validated outcome items, distributed across three domains: (1) Moral Competence (outcomes at the individual level), (2) Moral Teamwork (outcomes at the group level), and (3) Moral Action (outcomes at the case level). Each item assesses the degree to which the respondent agrees with it. The subdomains within the first domain, Moral Competence, are 'moral sensitivity' (participant's recognition of the ethical dimension of a case and awareness of the perspectives of others), 'analytical skills' (participant's ability to identify values at stake and provide arguments for and against different action courses), and 'virtuous attitude' (participant's ability to listen and courage to speak). The second domain, Moral Teamwork, includes the subdomains 'open dialogue' (participants feeling free and being facilitated to express their viewpoints and respecting others' viewpoints) and 'supportive relationships' (participants sharing emotions and being motivated to support each other when dealing with moral dilemmas). The last domain, Moral Action, includes the subdomains 'moral decision-making' (participants deciding how to act in the dilemma case and basing that decision on moral arguments) and 'responsible care' (participants being responsive to patients' and their families' needs and values and being able to explain and justify their care decisions to them).

MK and MB jointly checked which outcome (sub)domain resembled the content of the closed evaluation questions used in the present study, in order to be able to interpretate the results in terms of these outcome (sub)domains, as elaborated upon in the discussion section.

## Results

### Participants

In April–May 2020, 14 community pharmacists (two males, 12 females), along with two facilitators, participated in two MCD sessions, comprising seven participants (including the presenter) per session. These pharmacists were practising in different regions of the Netherlands, with an average work experience of 12 years in community pharmacy (range: 2–36 years). Their average age was 36 years (range: 26–63 year).

### Two MCD sessions

Each session took 2 h. In order to illustrate the dilemma method and the qualitative results of its methodical steps within the image-, judgement- and decision-making phases, only one of the two MCD sessions is presented along with relevant participants’ quotes.

### Phases 1–2: from image formation to judgement formation

In steps 1–3, participants grasped that the presenter’s dilemma centred on being unable to advise a patient's wife about double anticoagulant medication due to a lack of indication knowledge. The presenter presented two action options: not dispensing (option A) or dispensing (option B) the double anticoagulant medication.

Throughout the dialogue and factual questioning in various steps, it became apparent that the presenter felt the dilemma most acutely in her communication with the wife. This was evident when the wife shared concerns about her husband's health.

‘The wife told me about her husband’s suffering. I had doubts about his double anticoagulant therapy, as I was unsure about the indication and could only reach the initial prescriber after a few days. She relied on my knowledge and expertise to alleviate her concerns and sought advice on the most suitable medications for her husband.’ (Presenter P-MCD1, step 2)

The moral question central to the presenter’s dilemma, which she aimed to address in this MCD session, evolved in step 4 from ‘*Should I advise the wife of this patient now directly to continue using the double anticoagulant therapy in this situation?*’ (presenter P-MCD1) to ‘*Do the concerns of the wife justify stopping one of the two anticoagulants?*’ (participant W-MCD1). Step 5 further solidified the image of the presenter’s dilemma by incorporating the perspective of the geriatrician in the rehabilitation centre.

‘*He [the geriatrician] had to say that the double anticoagulation is right to continue because otherwise others would think of him that he had acted wrongly with continuing it all six weeks the patient stayed in the rehabilitation centre without knowing the indication.’* (Participant Z-MCD1, step 5)

In step 6, the participants formulated four alternative action options: (C) ‘*I advise the patient to contact the surgeon himsel*f’ (participant W-MCD1), (D) ‘*I only stop the dabigatran and advise to continue the acetylsalicylic acid*’ (participant V-MCD1), (E) ‘*I contact the patient myself*’ (participant Z-MCD1) and (F) ‘*I contact the alternate specialist in the hospital again*’ (participant T-MCD1). Alternative action option E shed new light on answering the presenter’s moral. The presenter initially hadn't considered option E, asserting that she knew the patient's recent history well and believed contacting him wouldn't yield new information. This reasoning became a crucial element in the subsequent decision-making phase.

In step 7, all participants gave their judgement with arguments for their chosen course of action. The dialogue then revolved around two contrasting judgements (Table 3).

**Table 3:** Judgement formation moral case deliberation session 1 (MCD1): Step 7 – Contrasting choices of action and moral considerations of participants X-MCD1 and Y-MCD1

Participant X-MCD1	Participant Y-MCD1
(a) It is morally justified that I choose option … (A, B or an alternative)	(a) It is morally justified that I choose option … (A, B or an alternative)
*‘I firstly choose option B: I contact the replacement physician and thereafter dispense the double anticoagulant medication.’*	*‘I firstly choose option E: I contact the patient and thereafter dispense the double anticoagulant medication.’*
(b) Because of…. (which value or norm?)	(b) Because of…. (which value or norm?)
*‘Reliable and caring, I want confirmation from the physician.’*	*‘Commitment to the well-being of the patient, I want to know the perspective from the patient himself, and not from what I think of the situation, or what the physician or patient’s wife think of the situation.’*
(c) Despite of…. (which value or norm?)	(c) Despite of…. (which value or norm?)
*‘Continuation of possibly incorrect double anticoagulation for a number of days with bleeding risk.’*	*‘I don't know to what extent this man is actually competent and accountable. Perhaps he is suffering from dementia and pretends to be better than reality or does not actually have an opinion. When I stop or start the anticoagulant unjustly, I might not be acting in the right way.’*
(d) How can you limit the disadvantages of your choice mentioned under c?	(d) How can you limit the disadvantages of your choice mentioned under c?
*‘I would inform the wife of the patient about the signs of oral anticoagulant toxicity such as bleeding and agree when she should call.’*	*‘By formulating questions in a careful manner, for example by saying: ‘I would like to ask you some more information, because you are the one who stayed in the rehabilitation centre.’*
(e) What do you need to act according your answer under ‘a’?	(e) What do you need to act according your answer under ‘a’?
Not answered in the session	Not answered in the session

### Phase 3: decision-making

The deliberation focused on the moral arguments put forth by four participants who opted for action option B (*‘I dispense the double anticoagulant therapy as per prescription and contact the specialist on Tuesday’*) and two participants who favoured alternative action option E (*'I first contact the patient myself*'). The divergence in moral arguments centred on the importance of the pharmacist obtaining the patient's perspective directly to offer advice to the wife about the medication.

*‘We always feel ourselves the home pharmacist of the patient; like the case presenter says, she knows the family, she knows the couple, but that can sometimes be a trap. So if I have the possibility to talk to the patient, then I prefer that action I want to assess the bleeding risk from my own observation . . . What are the patient’s experiences? . . . on the basis of that information, I choose the next action.*’ (Participant Z-MCD1)

The presenter realised the relevance of these viewpoints:

‘*Maybe the wife’s worries aren’t justified. Yes, if I had talked to the patient, if he was open to that, then you would get a more background and might be able to substantiate better the next action.*’ (Presenter P-MCD1)

As a result of the deliberation, the presenter and two other participants reconsidered their initial judgments from step 7 and shifted to alternative action option E. Consensus emerged that contacting the patient was deemed crucial in addressing the presenter’s moral question.

### Participants’ evaluation of the MCD session

Quantitative results, collected through the closed questions of the adapted Maastricht evaluation questionnaire, include participants' evaluations of both MCD sessions. These results are presented in Tables 4 and 5 (columns 2 and 3). Column 4 of these tables contain relevant participants’ quotes. These quotes were selected from either free text fields of open questions in the questionnaire (participants were given a number since the questionnaire was anonymously answered) or from the transcribed texts of the MCDs’ step 10 where participants evaluated the deliberation (participants were given a letter combined with MCD1 or MCD2 if they participated in the first or the second MCD session).

**Table 4:** Closed questions of the adapted Maastricht evaluation questionnaire [12] used to evaluate the moral case deliberation (MCD) session with community pharmacists anonymously (N = 14)

(1)	(2)	(3)	(4)	(5)
	Yes	No	Participants’ quotes selected from the free text fields of open questions in the questionnaire (participant with a number) or from the transcribed MCD session (participant or presenter with a letter)	Euro-MCD 2.0: outcome domains at individual level, group level or case level (main outcome domain; subdomain thereof)
1. Have you ever participated in an MCD session before?	3	11		Not recognised in a domain
2. Was the MCD session useful?	14	0		Not recognised in a domain
3. Have you changed your vision on how to approach the dilemma case due to the MCD session?	6	8	Presenter P-MCD1: *I learned that in my case contacting the patient would have been a very good action because the person often has just a slightly different view on the case. I have not thought about it during that moment. In this case it was luck that the patient later on confirmed the worries of the wife. But in general this is often not the case*	Individual level (moral competence; subdomain: moral sensitivity)
			Participant 7: *The MCD session led to new insights, and you realise that sometimes you want to find a solution too quickly*	
			Participant 1: *I didn't see moral dilemmas before, but because of the structured method it offers more insight into a past situation and helps you for the next time such a situation occurs*	
4. Have you become more aware of your own arguments and their underlying values due to the MCD session?	12	2	Participant 4: *Awareness. Interesting to see how others would handle a situation. You often take moral decisions in which you weigh risks, *etc*.... you are not aware on the basis of which professional values you are doing that*	Individual level (moral competence; subdomain: moral sensitivity)
			Participant W-MCD1: *The professional values are inside of you but during practice you are not aware of them. The MCD brought this for me. This case I would also discuss with my colleague in the pharmacy, hence MCD fits what you do in daily practice*	
5. Have you become more aware of the arguments with underlying values of other participants due to the MCD session?	14	0	Participant 9: *You learn from each other's input. Not only about the case itself, but also how colleagues are organized as care providers and how care is organized in their practice (roughly)*	Individual level (moral competence; subdomain: moral sensitivity)
			Participant K-MCD2: *Step 8 (dialogical inquiry) went a little too fast. I would have liked to hear all participants’ considerations, as these were asked in step 7*	
6. Would you like to participate in MCD sessions more often?	13	1		Not recognised in a domain
7. Do you think your fellow pharmacists are open to participating in an MCD session?	10	4		Not recognised in a domain

**Table 5:** Statements in the adapted Maastricht evaluation questionnaire [12] used to evaluate the moral case deliberation (MCD) session with community pharmacists, about the facilitator, participants and support from the MCD session (N = 14)

(1)	(2)	(3)	(4)	(5)
	Disagree	Agree	Participants’ quotes selected from the free text fields of open questions in the questionnaire (participants with a number) or from the transcribed MCD session (participants with a letter)	Euro-MCD 2.0: outcome domains at individual level, group level or case level (main outcome domain; subdomain thereof)
*The facilitator:*				
1. Facilitated an open atmosphere where I felt safe to say something	0	14		Group level (moral teamwork; subdomain: open dialogue)
2. Encouraged participants to keep asking each other open (factual) questions instead of judging	0	14	Participant 10: *Useful to deliberate such moral dilemmas with colleagues, other insights contribute to your own careful handling*	Group level (moral teamwork; subdomain: open dialogue)
3. Ensured that the focus remained on the moral core question during the deliberation	1	13		Group level (moral teamwork; subdomain: open dialogue)
4. Encouraged the participants to critically reflect	0	14		Group level (moral teamwork; subdomain: open dialogue)
5. Ensured that participants always substantiated opinions with arguments and in relation to the facts	0	14		Group level (moral teamwork; subdomain: open dialogue)
6. Encouraged the participants to investigate possible mutual similarities and differences in arguments	2	12	Participant 6: *There are several ways to reach the same decision. There is not necessarily a right or wrong way. It is useful to see a different approach, which you would not immediately choose yourself*	Group level (moral teamwork; subdomain: open dialogue)
			Participant 3: *I value MCD in order to know the perceptions from other colleagues*	
*The participants:*				
7. Respected each other’s arguments/viewpoints	0	14		Group level (moral teamwork; subdomain: open dialogue)
8. Were interested in the theme of the dilemma case	0	14		Not recognised in a domain
*The MCD session supported participants in:*				
9. Recognising the moral problem (i.e. the moral dilemma) in the dilemma case	0	14		Individual level (moral competence; subdomain: moral sensitivity)
10. Determining the necessary relevant (pharmaceutical) information in relation to the moral problem	2	12	Participant 5: *Try to gather as much background information as possible before you form your own judgement*	Individual level (moral competence; subdomain: analytical skills)
11. Recognising conflicting (professional) values in the action options of the dilemma	0	14	Participant 8: *The case indicated well that every obvious action option has disadvantages in the quadrilateral patient-treating physician–pharmacist–primary care physician*	Individual level (moral competence; subdomain: analytical skills)
			Participant 2: *Also looking to the disadvantage of a choice is not in my normal course of action*	
12. Determining the wishes/expectations of the patient (patient perspective)	1	13	Participant Y-MCD1: *I see the bleeding risk, but I want to assess that from my own observation... What are the patient’s experiences?... And then on the basis of that information, I can choose the next action*	Case level (moral action; subdomain: responsible care)
13. Determining the perspectives of all parties involved in the dilemma	1	13	Participants 7: *It is good to sometimes consciously distance yourself from a situation and look at the various stakeholders and perspectives with a helicopter view, in order to be able to make a well-considered decision*	Individual level (moral competence; subdomain: moral sensitivity)
14. Expressing arguments for and against the action the pharmacist chose to handle the dilemma	0	14	Participant 6: *There are several ways to reach the same decision. There is not necessarily a right or wrong way. It is useful to see a different approach, which you would not immediately choose yourself*	Individual level (moral competence; subdomain: analytical skills)
			Participant 11: *In de MCD you weigh the pros and cons of a choice more consciously. In daily practice, this happens more on autopilot, so the method of moral deliberation could therefore be useful*	
15. Morally justifying the final decision the pharmacist made to handle the dilemma	0	14	Participant M-MCD2: *Ultimately, it helps to choose a more well-founded conclusion and course of action. I think it is a nice method to be involved in the profession*	Case level (moral action; subdomain: moral decision-making)
16. Identifying the decisive (professional) value	0	14		Case level (moral action; subdomain: moral decision-making)

Tables 4 and 5 (columns 2 and 3) reveal that ten out of the 14 pharmacists encountered moral dilemmas in their practice on a daily or weekly basis, while four faced them on a monthly basis. Seven pharmacists almost always discussed these dilemmas with colleagues, and seven sometimes engaged in such discussions.

All 14 participating pharmacists found their session useful and expressed interest in the presented dilemma themes. Although three participants had prior experience with moral case deliberation, nearly all participants (n = 13) would like to participate again, with ten stating that their colleagues might also find MCD interesting.

Overall, the participants felt well-supported by the MCD (Table 5; 12 to 14 participants). They were able to identify moral dilemmas and conflicting professional values, gain awareness of their own and others' arguments and underlying values, and understand individuals’ perspectives involved in the dilemmas. Almost all participants felt comfortable expressing themselves, received support in dialogical skills like reflection and asking open questions, and were better able to justify their final decisions based on professional values.

Regarding the impact of the MCD on their view of the dilemma case, six participants reported a change in their perspective.

## Discussion

### Statement of key findings

This study highlights that moral case deliberation (MCD) holds the potential to enhance moral reflectivity among community pharmacists facing moral dilemmas.

### Strengths and weaknesses

Firstly, the study employed only two MCD sessions. Second, the MCD sessions were conducted online via videoconferencing due to COVID-19. While this offers implementation advantages such as enabling pharmacists from various geographical locations to join without travel constraints and potentially fostering more candid discussions, there are disadvantages, including the potential for missing body language cues and challenges in addressing heavy emotions related to the dilemma case [29]. Future research should consider qualitative and quantitative studies to expand upon these initial findings [21, 30]. Further research, possibly involving a series of MCD sessions, could provide broader insights into MCD's effects on moral reflectivity, particularly as pharmacists become more familiar with each other and the method, fostering mutual trust and openness on sensitive issues [12, 14]. A strength of the study lies in ensuring data trustworthiness through triangulation [20, 30], using two data sources: audio recordings of deliberations and an anonymous online questionnaire. However, self-motivation of participating pharmacists could have influenced the positive results.

### Interpretation

As mentioned in the method section, for interpretation of the results the validated MCD outcome domains of the European Moral Case Deliberation Outcomes Instrument (Euro-MCD 2.0) [28] were utilized.

In the realm of ethical competences at the individual level (Euro-MCD 2.0, (sub)domain 1), both moral sensitivity and analytical skills prove equally pertinent for pharmacists grappling with moral dilemmas. The findings from the study indicate that pharmacists experienced an enhancement in their moral sensitivity through MCD, particularly in the identification of moral dilemmas and the associated value conflicts. MCD’s structured process, involving guided deliberation and consideration of various perspectives on the dilemma, heightened pharmacists' awareness of the role of professional values in ethical decision-making. Additionally, MCD contributed to the development of pharmacists' analytical skills, enabling them to evaluate conflicting action courses, values, and perspectives more effectively. The structured nature of MCD, spanning from joint image formation, through judgment to decision-making, and emphasizing 'Socratic' skills like open questioning and delayed judgment, facilitated pharmacists in understanding the impact of various courses of action or their absence on the quality of pharmaceutical care and patient well-being. Through dialogue, some participants even altered their approach to the dilemma case, providing explicit arguments rooted in professional values that might have remained tacit knowledge without MCD. The study suggests that MCD serves as a valuable method for cultivating reflective moral competences crucial for pharmacists’ professional roles, as defined in frameworks such as the Canadian Medical Education Directions for Specialists (CanMEDS) [31, 32]. For instance, in their role as pharmaceutical experts, community pharmacists are expected to deliver effective and ethically responsible care in accordance with the profession’s ethical standards.

In the Netherlands, the post-graduate pharmacy education program aligns with the CanMEDS framework. The program incorporates Entrustable Professional Activities (EPAs) along with accompanying assessment instruments designed to measure pharmacists' development of the competences needed for those professional activities in community pharmacy [31]. However, currently there is no 'assessment' instrument available specifically designed to measure or monitor development of community pharmacists’ reflective moral competences. To address this gap, moral case deliberation could be instrumental.

Most moral case deliberation studies primarily focus on the structural implementation of the method in clinics or organizations, often within multidisciplinary teams [6, 8, 10, 12], resulting at the group level in stronger emotional connections among team members (Euro-MCD 2.0, (sub)domain 2). Assessing the impact of MCD on supportive relationships among pharmacists as a group in this study may be premature: the sessions were conducted only once for two distinct groups of pharmacists, all working in different pharmacies. The structure of local Dutch community pharmacies, where only a few pharmacists are typically present, limits opportunities for discussing moral dilemmas and sharing perspectives. Despite this, participants in the two MCDs felt supported by understanding how other pharmacists would handle similar dilemmas in their respective pharmacies. Deliberation enabled them to compare their practices with those of their peers, leading to changes in their initial clinical and moral judgments and decisions regarding the dilemmas. Therefore, support was derived from the comparison, revealing new knowledge about their own professionalism. This seems relevant taking in view the frequency and perceived burden and influences of ethical conflicts on decision-making in pharmacy practice [33] and high prevalence of burnouts among pharmacists [34]. Further research is therefore needed to determine if pharmacists also gain deeper emotional connections and support through moral case deliberation to better cope with moral dilemmas.

MCD outcomes at the case level (Euro-MCD 2.0, (sub)domain 3) showed that MCD helped pharmacists prioritize professional values when justifying their decisions regarding moral dilemmas, leading to more informed choices for patient care. In everyday pharmaceutical care practice, time constraints often limit such rational deliberation and reflection. Combining the competences developed at the individual and group level, pharmacists may approach their pharmaceutical care decisions more consciously and explicitly.

### Further research

The study suggests integrating moral case deliberation into professional ethics pharmacy education. Further research is recommended for continued empirical evaluation with community pharmacists. It would be interesting to understand its value as well at the level of master pharmacy students in order to prepare them with moral reflectivity skills to be used later in their professional life in community pharmacy.

## Conclusion

This study demonstrates the educational value of the dilemma method in moral case deliberation (MCD), guided by a trained facilitator. MCD enhances community pharmacists’ moral reflectivity, enabling them to address moral dilemmas in community pharmacy practice. Using a bottom-up approach rooted in real life experiences, MCD fosters collaborative critical reasoning and morally sound resolutions, supporting pharmacists’ professionalism and ethical decision-making and improving pharmaceutical care.

## Supplementary Information

Below is the link to the electronic supplementary material.

Supplementary file1 (DOCX 25 KB)
