# Supplementary material for: Fostering moral reflectivity in community pharmacists through moral case deliberation using the dilemma method
Source: Int J Clin Pharm. 2025 Jan 4;47(3):676–87. doi: 10.1007/s11096-024-01854-3 (PMC12125064; doi:10.1007/s11096-024-01854-3)
Supplement: Supplementary file 1 — Supplementary file1 (DOCX 25 KB) [file 11096_2024_1854_MOESM1_ESM.docx]

**Supplementary material**

| **Professional core values customised for community pharmacy [25]** |
| --- |
| **Commitment to the patient’s well-being** |
| The pharmacist is committed to the patient’s well-being. This commitment includes respecting the patient’s preferences and values and subsequently facilitating shared decision-making. The pharmacist respects the patient’s right to self-determination. |
| **Pharmaceutical expertise** |
| The pharmacist is a competent expert who helps patients and doctors optimize the effective and safe use of medicines. The pharmacist’s expertise emanates from speciﬁc knowledge of (patho)physiology, pharmacotherapy, pharmacokinetics, pharmacodynamics, pharmaceutics and health psychology. |
| **Reliable and caring** |
| Medicines can be highly effective but at the same time carry risks of causing harm. Quality assurance by the pharmacist is therefore crucial. The pharmacist acts meticulously and carefully (e.g. compounding or dispensing medicines, counselling patients, monitoring medicine use and keeping patient records). The pharmacist maintains a relationship of trust with the patient. Moreover, the pharmacist respects the patient’s conﬁdentiality. The pharmacist acts reliably within collaboration with other health professionals. |
| **Responsibility to society** |
| The pharmacist is responsible for the societal consequences of his or her actions. In order to maintain patients’ and the public’s trust in the pharmacy practice and the healthcare system, the pharmacist acts transparently and treats patients equally. The pharmacist guarantees access to pharmaceutical care and its continuity through collaboration with other health professionals. |
| **Professional autonomy** |
| The autonomy of the pharmacist stands in an independent relationship with that of other care professionals, healthcare insurers and the patient’s right of self-determination. The pharmacist is responsible for his or her decisions and adheres to the frameworks established by society. The pharmacist uses pharmaceutical judgment whilst maintaining a balance between commitment to the patient and the socially responsible course of action. |
